# Memorizing Doses

**Published:** 1887-01

**Authors:** 


Memorizing Doses.—Dr. G. A. Wiggins, of Philadelphia, (*Med. World*, Aug., 1886,) gives some general rules, with their exceptions, which are thoroughly reliable :

1. The dose of all infusions is 1 to 2 ozs., except infusion of digitalis, which is 2 to 4 drs.
2. Dose of all poisonous tinctures is 5 to 20 minims, except tincture of aconite, which is 1 to 5.
3. Dose of all wines is from ½ to 1 fl. dr., except wine of opium, which is 5 to 15 minims.
4. Of all poisonous solid extracts you can give ½ gr., except extract of calabar bean, which is 1-16 to ¼ gr.
5. Dose of all dilute acids is from 5 to 20 minims, except dilute hydrocyanic acid, which is 2 to 8 minims.
6. Dose of all aquæ is from 1 to 2 ozs., except aqua lauro-cerasus and aqua ammonia, which are 10 to 30 minims.
7. Of all syrups, you can give one drachm.
8. Dose of all mixtures is fom ½ to 1 fl. oz.
9. Dose of all spirits is from ½ to 1 fl. dr.
10. Dose of all essential oils is from 1 to 5 minims.

